# Reversed shoulder arthroplasty leads to significant histological changes of the deltoid muscle: a prospective intervention trial

**DOI:** 10.1007/s00402-020-03503-6

**Published:** 2020-06-11

**Authors:** Matthias Koch, Christian Schmidt, Maximilian Kerschbaum, Tobias Winkler, Christian G. Pfeifer, Stefan Greiner

**Affiliations:** 1 Department of Trauma Surgery, University Medical Centre Regensburg, Franz-Josef-Strauss-Allee 11, 93053 Regensburg, Germany; 2 Orthopaedic Department, Center for Musculoskeletal Surgery, Charité-Universitätsmedizin Berlin, Berlin, Germany; 3 Sporthopaedicum Regensburg/Straubing, Hildegard-von-Bingen-Str. 1, 93053 Regensburg, Germany

**Keywords:** Shoulder joint, Rotator cuff, Cuff tear arthropathy, Reverse shoulder arthroplasty, Deltoid muscle, Histological changes of the deltoid muscle

## Abstract

**Introduction:**

Reverse shoulder arthroplasty (RSA) shows promising short- and mid-term results in cuff tear arthropathy. However, functional impairments are described in long-term findings. Micromorphological changes in the periarticular musculature could be in part responsible for this, but have not yet been analysed. Thus, histological changes of the deltoid muscle and their association to the functional outcome were evaluated in this study.

**Material and methods:**

A total of 15 patients treated with RSA were included in this prospective study. Functional outcome was assessed using the Constant Score (CS) and the DASH (disabilities of the arm, shoulder and hand) Score before RSA and after a mean follow-up of 12 months. Deltoid muscle biopsies were harvested intraoperatively and 12 months postoperatively. Mean deltoid muscle fibre area (MMFA) was calculated histologically after haematoxylin–eosin staining.

**Results:**

Postoperative shoulder function significantly improved within 12 months (CS: Δ 37.4 ± 22.6, *p* = 0.001; DASH: Δ 27.1 ± 29.1, *p* = 0.006). The MMFA significantly decreased (*p* = 0.02), comparing the results from the intraoperative biopsy (MMFA: 8435.8 µm^2^, SD ± 5995.9 µm^2^) to the 12 months biopsy (MMFA: 5792. µm^2^, SD ± 3223.6 µm^2^). No correlation could be found between the functional score results and MMFA.

**Conclusion:**

Signs of deltoid muscle changes in terms of a reduced MMFA can be detected 1 year after RSA and thus already a long time before long-term functional impairments become apparent. Further studies with larger patient series and longer follow-up periods as well as extended histological assessments and simultaneous radiological examinations are required.

## Introduction

Grammont et al. developed the first reverse shoulder prosthesis (RSA) at the end of the 1980’s [1]. Positioning the convex part of the shoulder on the glenoid and the concave matching surface on the humerus in patients with absent rotator cuff characterized this non-anatomical approach [2]. Thereby, the biomechanical joint composition in terms of medializing of the glenoid rotation centre as well as lengthening of the deltoid lever arm by distalizing its humeral insertion was changed. This enables the deltoid muscle to partially cover the functionality of the rotator cuff [3–5]. Up to now, it is still the principal of the following advanced models of reverse shoulder prosthesis. While the first models were associated with a short life period and a high complication rate [1], the current short- and mid-term results in patients with cuff tear arthropathy are promising [1, 6–8]. However, long-term results show a loss of function even without prosthesis loosening [9–12]. Subsequently, Greiner et al. investigated the muscular status after RSA on a radiological basis [4]. They showed a time-dependent correlation of the amount of degenerative changes within the deltoid muscle and follow-up. Furthermore, Fischer et al. demonstrated a positive correlation between the deltoid muscle perfusion and shoulder function after RSA using dynamic contrast-enhanced ultrasound [5]. In elastography testing, they also showed a higher stiffness in the deltoid muscle of the operated side in comparison to the contralateral side. Overall, functionally relevant axillary nerve injuries were excluded before. Also Li et al. emphasize the impact of the deltoid muscle assessed by electromyography on the post RSA clinical outcome [13]. Both, shoulder strength as well as active range of motion positively correlated with the preoperative electromyography activity of the deltoid muscle.

Based on the increased load on the deltoid muscle after RSA and the initial postoperative functional improvements, muscular hypertrophy of the deltoid muscle in terms of an increased mean muscle fibre area (MMFA) has to be assumed regarding the findings of Fischer et al. who described an increased deltoid caliber 6 months after RSA based on contrast-enhanced ultrasound analysis [14]. However, according to the current literature, there is no histological analysis of the deltoid muscle fibres after RSA. So, the aim of this study was to analyse postoperative changes of the deltoid muscle in comparison to the perioperative muscle status on a histological basis and with respect to the clinical outcome in a cuff tear arthropathy population.

## Material and methods

### Patient selection

The study was approved by the local Institutional Review Board (Code: EA1/072/09) and written consent amongst others concerning participation, harvesting biopsies intraoperatively and at 12 months follow up as well as publication of the data was collected for each patient. 18 patients affected by symptomatic cuff tear arthropathy (CTA) and consecutively the indication for implantation of reverse shoulder arthroplasty were included in the present, prospective intervention trial in the period of July 2010 to September 2014. Symptomatic CTA was defined as CTA classified more than grade 2 according to the Hamada classification [15], as well as persisting severe pain after failed conservative treatment of a minimum of 6 months and additionally limited active range of motion (abduction and flexion less than 90°). Further inclusion criteria, the availability of the complete data of the CS, DASH score as well as complete histological analysis of intra- and postoperative deltoid muscle biopsies.

Exclusion criteria were defined as patients younger than 65 years or with relevant glenoid bone loss. Also, patients with post-traumatic conditions or prior open surgery using a delta split approach were excluded in this study.

### Implantation of the reverse shoulder replacement

All operations were performed by one surgeon, always using the same approach, technique and prosthesis. In all patients, the reverse shoulder arthroplasty was performed using the deltoid-pectoral approach and dissection or, if possible, consecutive re-fixation of the subscapularis tendon. In these cases, the subscapularis tendon was re-fixed using number 2 fibre wire. Three to four Mason Allan stiches were passed through the subscapularis tendon stump and following sutures were passed transosseously through the area of the minor tuberosity and the bicipital groove.

The Aequalis Reversed II Shoulder Prosthesis (Wright Medical System) with a cemented humeral stem and 36 glenosphere was used in all patients.

All patients underwent the same aftercare including a shoulder abduction sling for 3 weeks with immediate passive abduction and flexion in 60° internal rotation position and no external rotation for 6 weeks.

### Biopsy of the deltoid muscle

Muscle samples of the deltoid muscle were harvested intraoperatively after the implant was put in place using a sterile 5 mm muscle punch (Bard Magnum, Bard Biopsy Systems, Tempe, USA) The biopsy was taken percutaneously a distance 7 cm below the antero–lateral acromion edge to harvest representative deltoid muscle of the acromial part.

According to the written consent and explanations before patients’ inclusion in this study, after 12 months, patients were asked to sit down in a beach chair position with the arm at the side, comparable to the positioning during the operation. At the same site, a distance of 7 cm below the antero–lateral acromion edge the skin was infiltrated with local anaesthetics (Xylonest 2%) and a 5 mm skin incision was performed. Again, the 5 mm muscle punch was used for taking the muscle biopsy perpendicular to the fibre direction. The wound was closed using steristrips. The muscle specimens were embedded in TissueTek (Sakura Finetek Germany, Staufen, Germany), and frozen in nitrogen cooled iso-methylbutan (Sigma, Taufkirchen, Germany) for 2 min.

### Processing of the muscle samples and histological analysis

Cross-sectional cryosections of fine-needle biopsies (10 µm thickness) were produced using a cryostat cutter and fixated on an object slide before conventional haematoxylin–eosin staining was performed.

**Fig. 1:**
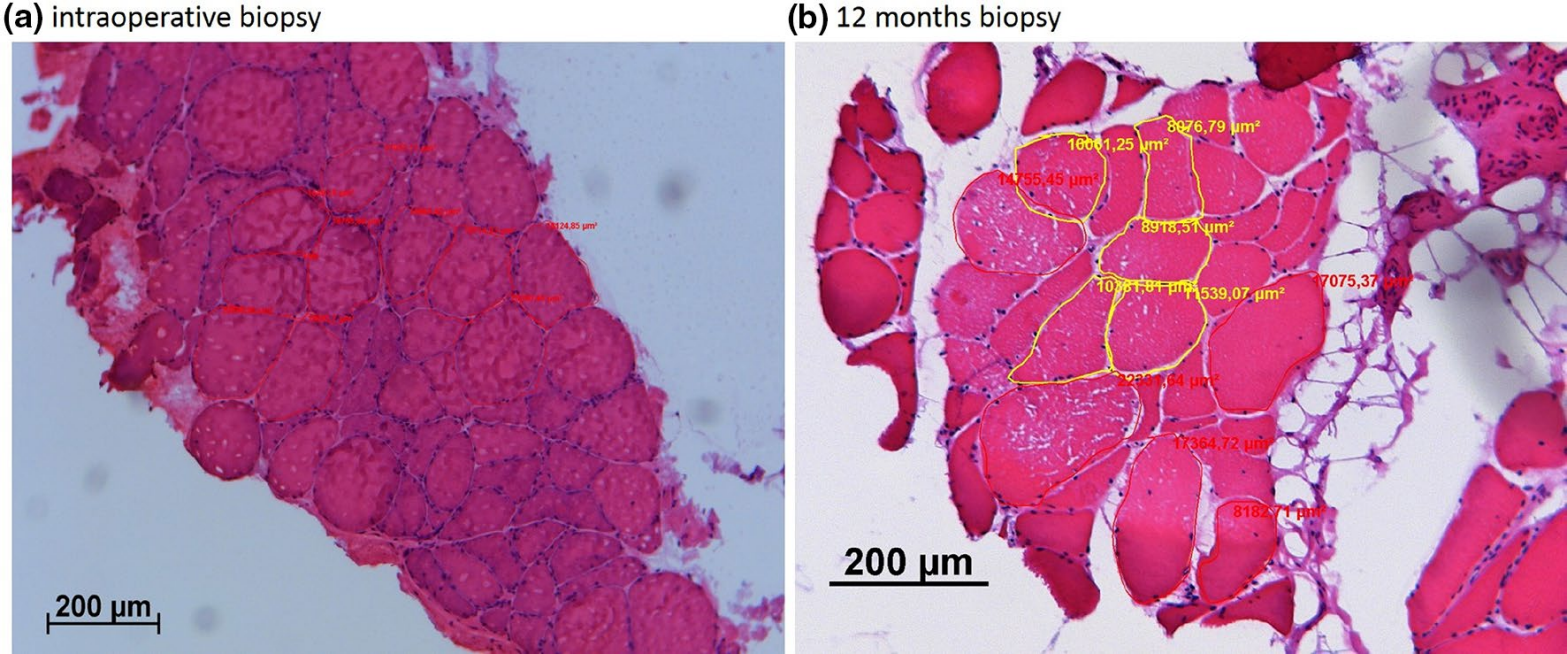
Software-based calculation of the mean muscle fibre area (MMFA) after muscle sample processing and haematoxylin–eosin staining based on ten randomly chosen different muscle fibre sections. **a** Intraoperative biopsy, **b** 12 months biopsy

Fibre diameters were measured on haematoxylin–eosin sections, measuring a minimum of 200 fibres per biopsy on representative areas of the muscle sample. All sections were analysed by a Leica DMRB light microscope (Leica, Wetzlar, Germany) equipped with an AxioCam MRc (Carl Zeiss, Goettingen, Germany). For calculation of the MMFA, ten randomly chosen representative high power fields of each muscle sample were analysed in a 400× enlargement (see Fig. 1). Image evaluation and MMFA calculation were performed with the software KS 400, Version 3.0 (Carl Zeiss, Goettingen, Germany).

### Subjective and functional outcome evaluation

Functional and subjective outcome was evaluated prior to the surgery and after a mean follow-up of 12 months using the DASH (disabilities of the arm, shoulder and hand) Score [16] and the Constant Score [17].

### Statistics

Statistical analysis was performed using the SPSS software version 23.0 (SPSS, Chicago, IL, USA) to determine relationships between variables. Spearman correlation test was used for quantitative data analysis. To determine whether data followed a Gaussian distribution, a Kolmogorov–Smirnov-test was conducted and the Wilcoxon test was chosen for qualitative analysis of the related groups. The significance level was set at *p* ≤ 0.05. The variables analysed included the pre- and postoperative Constant Scores (CS), the pre- and postoperative DASH Scores and the mean deltoid muscle fibre area at the time of RSA implantation and 12 months after RSA.

## Results

### General data

In this prospective study, a total of 18 patients fulfilled the inclusion criteria. Overall, ten patients were right-handed and eight patients were left-handed. In 11 patients, the dominant site was affected. 3 patients had to be excluded due to loss to follow-up. The included patients were aged between 67 and 85 years with a mean age of 75.4 years (SD ± 4.6 years). There were nine female and six male patients. No significant correlation between age, functional outcome and MMFA intra- and postoperative was found.

### Clinical outcome

**Fig. 2:**
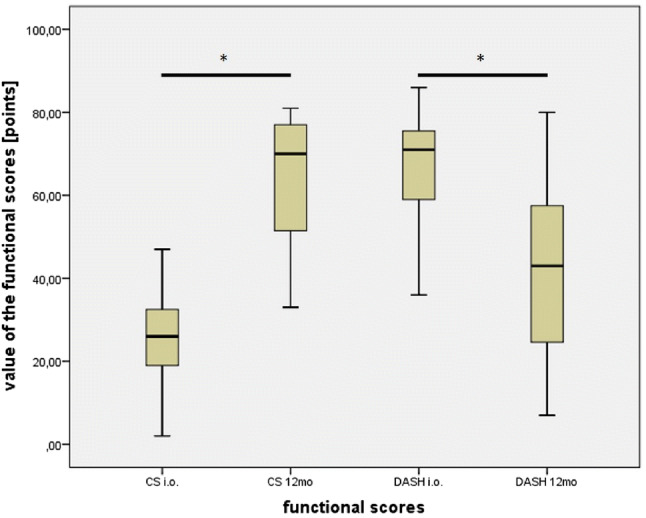
Quantitative evaluation of the functional outcome 1 year after RSA by CS preoperative and postoperative (= 12 months follow-up) as well as DASH Score preoperative and postoperative (= 12 months follow-up). Both scores show a significant gain of function within 1 year after RSA. Significance level: *p* < 0.05

Functional and subjective outcome was assessed preoperatively and at the 12 month follow-up. The mean absolute CS of all evaluated patients (*N* = 15) increased significantly from 25.4 points (SD ± 11.5 points) preoperatively to 62.8 points (SD ± 17.3 points) after reverse shoulder arthroplasty (*p* value = 0.001). The mean DASH-score significantly decreased 1 year after RSA from 67.3 points (SD ± 13.3 points) to 40.2 points (SD ± 22.8 points) (*p* value = 0.006) (see Fig. 2).

### Histological evaluation

**Fig. 3:**
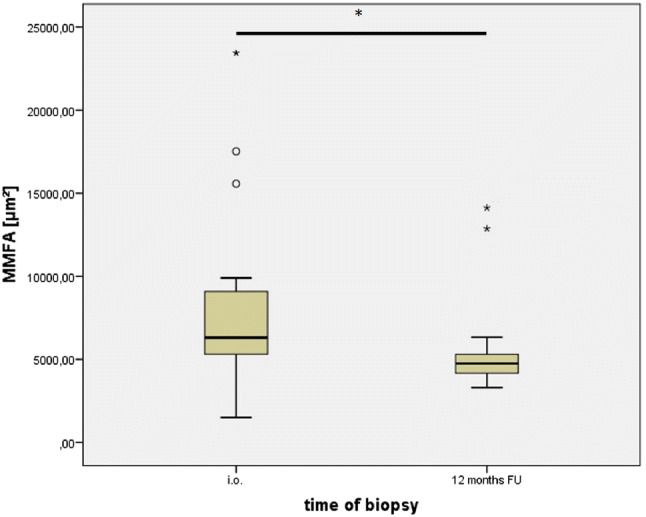
Quantitative evaluation of the mean deltoid muscle fibre area (MMFA, [µm^2^]) intraoperatively after RSA implantation as well as at 12 months follow-up. A significant reduction of the MMFA 12 months after RSA was detected. Significance level: *p* < 0.05. The ring describes spikes with 1.5–3-times distance to the 75% percentile and the star describes spikes with > 3-times distance to the 75% percentile. In both cases, the distance is defined as the interval between the 25% and 75% percentile

Biopsies of 18 patients were stained and analysed. 3 patients could not be included in the final evaluation due to non-representative 1 year postoperative fine needle biopsies. The histological evaluation of the mean deltoid muscle fibre area of the remaining 15 participants showed a significant reduction of the MMFA of around 30%. The MMFA decreased significantly from 8435.8 µm^2^ (SD ± 5995.9 µm^2^) intraoperatively to 5792. µm^2^ (SD ± 3223.6 µm^2^) (*p* value = 0.02) within 12 months (see Fig. 3). No significant correlation between the MMFA and pre- and postoperative score results were found.

## Discussion

The present study evaluated for the first time the effect of a reverse shoulder arthroplasty on the deltoid muscle on a histological basis. Clinical results after reverse shoulder arthroplasty for cuff tear arthropathy have consistently shown to be promising [6–8]. The initial gain of functional improvement after reverse shoulder arthroplasty bases on the ability of the deltoid muscle to partially cover the lack of rotator cuff function in cuff tear arthropathy. Due to the changed joint composition, the deltoid muscle is described as the main muscular motor for functional abilities [3–5]. Therefore, in the context of this new task and the associated enduring stress for the purpose of a continuous muscle loading, the consecutive hypertrophy of deltoid muscle fibers could be hypothesized after reverse shoulder arthroplasty [18, 19].

Surprisingly, the histological evaluation not only showed no hypertrophy, but also a significant reduction of the relative MMFA of—25.5% within 12 months after reverse shoulder arthroplasty. Various factors, such as postoperative immobilization of the index shoulder, postoperative lengthening of the deltoid muscle or perioperative modified perfusion have to be considered as possible explanations for this.

Postoperative immobilization is known to be associated with muscle atrophy and consecutively loss of function [20, 21]. Regarding the participants of this study, overall a significant improvement concerning the functional outcome within 12 months after reverse shoulder arthroplasty implantation was observed. These results also correlate with the current literature [1, 6–8]. Based on this fact, the reduction of the MMFA in the deltoid muscle may not be assessed as a residuum of the postoperative immobilization.

Also isolated reduction of the mean muscle fibre area due to deltoid lengthening by distalizing the insertion at the humerus after reverse shoulder arthroplasty implantation seems to be improbable. Both biopsies were performed after the implant was put in place. So, even the first deltoid muscle samples were harvested at a lengthened status.

However, in this context, a modified perfusion of the deltoid muscle has to be discussed. It is well known that stretching of the muscular tissue, as performed by reverse shoulder arthroplasty [5], is associated with an impaired perfusion and nutrition [22]. On the other hand, endurance activity of muscles, like in the case of the deltoid muscle as the functional main motor after reverse shoulder arthroplasty, is described to increase muscular microcirculation [23]. Concerning this issue Fisher et al. evaluated the deltoid muscle function by scoring and perfusion by contrast enhanced ultrasound (CEUS) and acoustic radiation force impulse (ARFI) in comparison to the non-operated site [5]. Overall, they found an impaired deltoid function and sonographic diminished perfusion as well as reduced elasticity of the deltoid muscle comparing the reverse shoulder arthroplasty on the operated site with the non-operated contralateral site. So, an anaerobic milieu in the deltoid muscle has to be assumed according to the enhanced demands and reduced perfusion. Fisher et al. further suggested that the reduced perfusion kinetics of the deltoid muscle at the operated site could cause the recruitment of fewer muscle fibres during exercise [5].

Regarding the long-term outcome, the loss of function in long term [9–12] remains a concern, although short- and mid-term clinical results after reverse shoulder arthroplasty for cuff tear arthropathy have consistently shown to be promising [6–8]. The reasons for this loss of function are not fully understood so far. Beside biomechanical reasons for the loss of function in the long-term, such as polyethylene wear and implant loosening, the functional integrity of the deltoid muscle might also be one possible reason. Li et al. found a positive correlation between preoperative electromyography activity of the deltoid and postoperative shoulder strengths as well as active range of motion [13]. Greiner et al. showed that degenerative changes of the deltoid muscle have an impact on the clinical outcome [4].

Thus, the functional loss in long-term outcome might be attributed to a kind of weariness of the deltoid muscle in terms of a slow progression of muscle atrophy and consecutive loss of function by a nutritional undersupply over time.

Nevertheless, this study has some limitations. No generalizing conclusion can be drawn due to the small study population as well as the histologically based calculation of the relative mean deltoid muscle fibres area only from the RSA operated site and at one follow-up time point. However, the present study shows for the first time histologically based changes in the deltoid muscle after reverse shoulder arthroplasty before functional impairments are detected. These findings suggest that muscle degeneration leading to an impaired functional long-term outcome starts at an early point of time. So, the hypothesis of deltoid muscle hypertrophy caused by the increased loading after RSA has to be queried.

Therefore, further studies with larger patient series are required. These studies should include functional scores, radiological examinations [Sonography (CEUS), MRI] as well as detailed histological assessments (muscle fibre type, mean muscle fibre area, count of fibres, fatty infiltration, vascular density and pH-metry) pre- and postoperatively and also at different time points during a longer follow-up period. Additionally, the contralateral site should be analysed as a control.

## Conclusion

The evaluation of the deltoid muscle after RSA showed a significant decrease of the MMFA within 1 year post RSA implantation. To this date, the functional gain based after RSA showed no correlation to the histological findings. Based on this facts, structural impairments seem to be present already a long time before long-term functional impairments become apparent. Further studies with larger patient series and longer follow-up periods as well as extended histological assessments and simultaneous radiological examinations are required.
